# Prophylactic and therapeutic activity of fully human monoclonal antibodies directed against Influenza A M2 protein

**DOI:** 10.1186/1743-422X-6-224

**Published:** 2009-12-21

**Authors:** Roger R Beerli, Monika Bauer, Nicole Schmitz, Regula B Buser, Myriam Gwerder, Simone Muntwiler, Wolfgang A Renner, Philippe Saudan, Martin F Bachmann

**Affiliations:** 1 Cytos Biotechnology AG, Wagistrasse 25, CH-8952 Schlieren, Switzerland; 2 Actelion Pharmaceuticals Ltd, Gewerbestrasse 16, 4123 Allschwil, Switzerland; 3 GlycoVaxyn AG, Grabenstrasse 3, 8952 Schlieren, Switzerland; 4 Biozentrum, University of Basel, Klingelbergstrasse 50/70, 4056 Basel, Switzerland

## Abstract

Influenza virus infection is a prevalent disease in humans. Antibodies against hemagglutinin have been shown to prevent infection and hence hemagglutinin is the major constituent of current vaccines. Antibodies directed against the highly conserved extracellular domain of M2 have also been shown to mediate protection against Influenza A infection in various animal models. Active vaccination is generally considered the best approach to combat viral diseases. However, passive immunization is an attractive alternative, particularly in acutely exposed or immune compromized individuals, young children and the elderly. We recently described a novel method for the rapid isolation of natural human antibodies by mammalian cell display. Here we used this approach to isolate human monoclonal antibodies directed against the highly conserved extracellular domain of the Influenza A M2 protein. The identified antibodies bound M2 peptide with high affinities, recognized native cell-surface expressed M2 and protected mice from a lethal influenza virus challenge. Moreover, therapeutic treatment up to 2 days after infection was effective, suggesting that M2-specific monoclonals have a great potential as immunotherapeutic agents against Influenza infection.

## Background

Influenza A virus still is a major cause of disease in humans, accounting for three to five million cases of severe illness and 250,000 - 500,000 deaths each year [1]. Efficient influenza A vaccines are available, which induce antibodies predominantly against the two major components of the virus membrane, hemagglutinin (HA) and neuramidase (NA). Protection is mediated primarily by neutralizing antibodies against HA [2,3]. Since HA undergoes continuous change due to mutations (antigenic drift), new antigenic variants of influenza A arise every year requiring constant update of the vaccines. Effective vaccination is further complicated by the occasional reassortment of the segmented viral genome leading to the replacement of HA or NA from one subtype by another subtype, a processs called antigenic shift [4]. Passive immunization with monoclonal antibodies (mAbs) targeting HA is very efficient [5-7], however, suffers the same disadvantages as the current vaccines due to antigenic shift and drift.

An ideal target for active and passive immunization strategies would therefore be a conserved viral protein. The matrix protein 2 (M2) fits the bill and has received considerable attention as a potential target against influenza infection over the past decades [8-23]. M2 is a tetrameric ion channel [24-26] which is involved in virus uncoating in the endosome and in virus maturation in the trans-Golgi network [27-29]. Its 23 amino acid extracellular domain has remained remarkably conserved in human influenza A virus isolates over the last hundred years [30], at least in part due to the fact that the M2 protein is co-transcribed with the matrix protein 1 (M1) [31,32]. Whereas M2 is abundantly expressed on infected cells, only very few M2 molecules are present in Influenza A virus membranes [23,26]. In accordance with this, current seasonal influenza vaccines do not induce a significant humoral resonse against M2, and M2 specific antibodies (administered intravenously or induced by active immunization) mediate protection not by neutralizing virions, but by eliminating infected cells by ADCC [15,22].

Passive immunization with monoclonal antibodies has several advantages over vaccination. In particular, it allows treating people which poorly respond to vaccines, such as the elderly, young children or immune compromised individuals. In addition, passive immunisation is the treatment option of choice in situations where rapid protection is crucial, such as for post-exposure treatment or prophylaxis for the acutely exposed. A number of M2 ectodomain (M2e)-specific mAbs have been reported to protect mice from a lethal challenge in a prophylactic setting [12,17,21-23]. While these mAbs include fully human antibodies derived from transchromosomic mice [22], no natural human M2e-specific antibodies have been reported to date. However, for application in human subjects, natural human antibodies are the preferred choice. In contrast to humanized and fully human antibodies derived from phage display or transchromosomic mice, natural human antibodies combine the advantage of minimal immunogenicity with the smallest possible off-target reactivity and toxicity. Furthermore, human derived antibodies have the advantage of having gone through the affinity maturation process, resulting in high affinity antibodies.

We recently described a novel method for the efficient isolation of antibodies from humans by mammalian cell display [33]. Here, we used this method for the isolation of natural human antibodies directed against M2e. We demonstrate that the antibodies bind M2 with high affinity and efficiently recognize M2 from a recently isolated H5N1 influenza A strain. The antibodies not only have potent prophylactic activities in a mouse model of Influenzy A infection, but also show efficacy in a therapeutic setting. Thus, the natural human antibodies described here have potential as immunotherapeutics against influenza infection.

## Results and Discussion

### Isolation of M2e-specific human monoclonal antibodies

Human mAbs were isolated by Sindbis-mediated mammalian cell display [33]. First, 334 M2e-specific B cells were isolated by FACS from the peripheral blood mononuclear cells (PBMCs) of an individual with high M2e titers, using the M2e consensus peptide (M2e-cons) (Table 1) conjugated to the virus-like particle Qβ as a bait. The immunoglobulin variable regions (VRs) of the heavy chains (HCs) and light chains (LCs) were then amplified by RT-PCR and assembled to single-chain antibody (scFv) coding regions. Two separate libraries, one encoding scFv antibodies with κ, the other with λ LCVRs, were cloned in the Sindbis cell surface display vector pDel-SP-TM [33]. The resulting scFv-κ and scFv-λ sublibraries consisted of 1.0 × 10^6 ^and 1.1 × 10^6 ^independent transformants, respectively. Since the library was derived from 334 cells, it may be expected that every possible combination of heavy and light chain is covered multiple times by the 2 million clones and, accordingly, that screening is likely to yield antibodies containing the natural heavy and light chain pairs. Recombinant Sindbis virus libraries were then generated with titers of 3.4 × 10^7 ^pfu/ml and 5.7 × 10^7 ^pfu/ml, respectively, and used to infect BHK cells at a low multiplicity of infection (MOI), to ascertain expression of a single antibody species per infected cell.

**Table 1:** M2e variants used in this study

M2 variant	Abbreviation	Subtype	M2e (2-24) Sequence^(2)^
Consensus^(1)^	M2e-cons	n/a	SLLTEVETPIRNEWGCRCNDSSD

A/VN/1203/04	M2e-VN	H5N1	SLLTEVETP**T**RNEW**E**CRC**S**DSSD

A/PR/8/34	M2e-PR	H1N1	SLLTEVETPIRNEWGCRCN**G**SSD

(1) M2e consensus sequence derived from H1, H2, and H3 subtypes of human Influenza A viruses.

(2) Variations from M2e consensus sequence are shown in bold [38].

**Figure 1:**
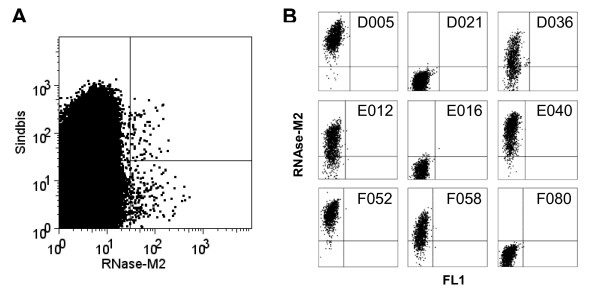
**(A) Isolation of BHK cells displaying M2e-specific scFv**. BHK cells were infected with the scFv-κ Sindbis virus library at an MOI of 0.1 and stained as indicated. Sindbis-positive cells displaying M2e-specific scFv in the upper right quadrant were sorted. Note that cells in the lower right quadrant (M2e-positive but Sindbis-negative) are infected with a replication-defective form of the virus. (B) Rescreening for M2e-specific antibodies. Two to three days after sorting, BHK cells in wells showing signs of viral infection were analyzed for binding to RNAse-M2e. Nine representative samples are shown, including cells showing strong binding (D005, E040, F052), intermediate binding (D036, E012, F058), or no binding (D021, E016, F080).

Cells were stained for cell surface expression of M2-specific scFv using RNAse-M2e-cons or Qβ-M2e-cons conjugates and subjected to FACS. No M2e-specific antibodies could be found on the surface of BHK cells infected with the scFv-λ sublibrary. In contrast, BHK cells infected with the scFv-κ sublibrary contained a substantial fraction of M2e-reactive cells (typically about 0.3% of infected cells). Three sorts were carried out with the scFv-κ library, two using Qβ-M2e-cons (not shown) and one using RNase-M2e-cons as bait (Figure 1A), leading to the isolation of 255 BHK cells each displaying an M2e-specific antibody. Single cells were sorted into wells containing BHK feeder cells and incubated for 2 to 3 days to allow for amplification of the corresponding Sindbis virus clone. In total, 201 of the 255 wells showed signs of viral infection and were reanalyzed for M2e binding, using RNase-M2e-cons as a bait (Figure 1B). 130 cells showing varying degrees of M2e binding were identified. Supernatants from wells containing infected BHK cells displaying highly M2e-reactive scFv were used to clone the corresponding scFv coding region by RT-PCR. The large number of specific antibodies isolated in a single screen not only illustrates the utility of mammalian cell display [33], but also makes it an attractive alternative to other methods, typically based on cloning of antibodies from single or amplified B cell clones [34,35], or from EBV-immortalized memory B cells [36].

Since the protective potential of M2e-specific antibodies depends on ADCC [15], the scFv antibodies were fused to the Fc region of mouse IgG2c (msFc-γ2c), allowing *in vivo *efficacy testing in a mouse model of influenza. All scFv-msFc-γ2c fusion proteins were expressed in HEK 293T cells, using an Epstein Barr virus (EBV)-based episomal expression vector. Binding of scFv-msFc-γ2c antibodies to M2e was confirmed by ELISA, using supernatants from transiently transfected cells (not shown). Clones D005, E040 and F052 were selected for further analysis, expressed in HEK-293T cells and purified by affinity chromatography. The three antibodies were expressed at high levels, with yields of 20 to 40 mg per liter. These yields are in the same range as the ones we previously reported for Qβ- and Nicotine-specific human antibodies [33], demonstrating that the mammalian cell display method consistently yields antibodies that are highly expressed in mammalian cells.

### In vitro analysis of M2e-specific monoclonal antibodies

**Figure 2:**
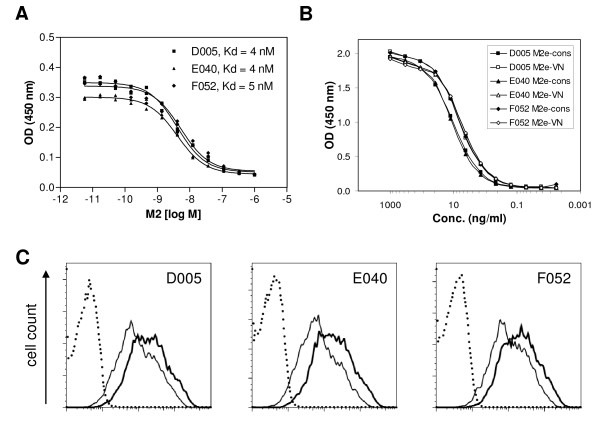
**Binding properties of M2e-specific scFv-msFc-γ2c antibodies**. (A) Determination of affinity. The dissociation constants (Kd) of antibody binding to M2e-cons peptide in solution was determined by Friguet ELISA. (B) Binding to H5N1 Influenza A derived M2e. Antibody binding to the indicated RNAse-M2e conjugates was measured at different concentrations by ELISA. (C) Binding to cell surface M2. L929-M2#E9 cells, a clone of L929 cells expressing full-length M2 derived from mouse-adapted H1N1 Influenza A PR/8/34, were stained with the indicated antibodies and analyzed by FACS. Bold lines, 0.5 μg/ml; solid lines, 60 ng/ml; dotted lines, secondary antibody alone.

We next determined the affinity of D005, E040, and F052 for M2e. To this end, the dissociation constants (Kd) of antibody binding to M2e-cons in solution were determined using an ELISA-based method [37] (Figure 2A). The scFv-msFc-γ2c antibodies were found to bind M2e-cons peptide with high affinity, with Kd values of 4-5 nM (Figure 2A).

In a next set of experiments the ability of the antibodies to cross-react with a peptide covering M2e from a recent Influenza A H5N1 isolate (A/VN/1203/2004) was assessed by ELISA. The sequence of the peptide used for coating (M2e-VN) is shown in Table 1. Thus, ELISA plates were coated with M2e-cons or M2e-VN peptide conjugated to RNAse A, and serial dilutions of the different scFv-msFc-γ2c antibodies were applied. Significantly, each of the antibodies bound both peptides to a similar extent, with EC50 values of 5-10 ng/ml (45-90 pM), indicating that they efficiently crossreact with H5N1-derived M2e.

Finally, binding of the antibodies to native, cell surface M2 was investigated (Figure 2C). To this end, a clone of L929 cells expressing full-length M2 derived from Influenza A/PR/8/34 (H1N1) was generated (L929-M2#E9). The sequence for the extracellular domain of the M2 is shown in Table 1 (M2e-PR). The cells were stained with increasing concentrations of D005, E040 or F052. A concentration-dependent staining of the cells was observed, demonstrating that each of the antibodies is capable of recognizing cellular, native M2 protein. In agreement with the ELISA results, the three antibodies showed similar binding to L929 cells expressing M2.

### Prophylactic activity of M2e-specific antibodies against influenza virus infection

**Figure 3:**
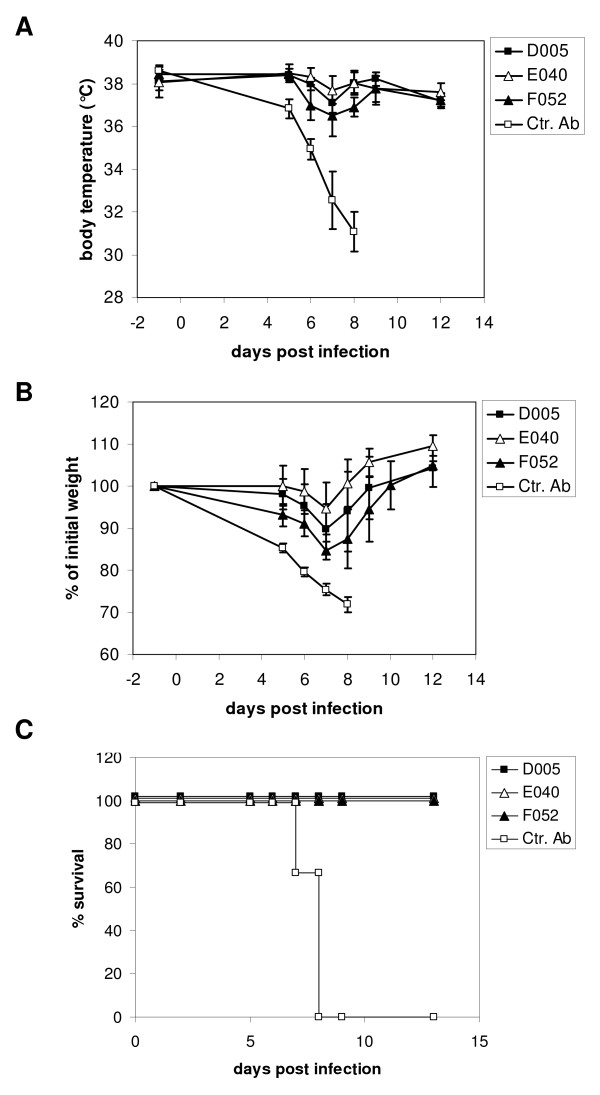
**Effect of M2-specific scFv-msFc-γ2c antibodies on Influenza-induced morbidity and mortality**. Mice were treated with 500 μg of the indicated antibody on day -2, infected with Influenza A virus PR8 on day 0, and body temperature (A), weight (B) and survival (C) were monitored over time.

We next explored the clinical potential of the antibodies by investigating their protective activity in an *in vivo *model of influenza A infection. This model reflects most aspects of Influenza infection in humans and is therefore routinely used to assess the efficacy of anti-viral agents. Groups of mice were passively immunized *i.p*. with 500 μg scFv-msFc-γ2c antibodies D005, E040 or F052, while a control group received the same amount of mouse IgG. Two days later, animals were infected intranasally with a lethal dose (4 × LD50) of mouse adapted (m.a.) influenza A/PR/8/34 and monitored for morbidity and mortality (Figure 3). Control mice treated with mouse IgG showed a dramatic drop in body temperature and substantial weight loss within few days after infection and and had to be euthanized on days 7 or 8 due to the severity of the symptoms (Figure 3C). In contrast, all animals that had been treated with M2-specific antibodies survived the lethal challenge, hardly developed any fever and only displayed a transient weight loss (Figure 3A and 3B).

### Titration of M2e-specific antibody D005

**Figure 4:**
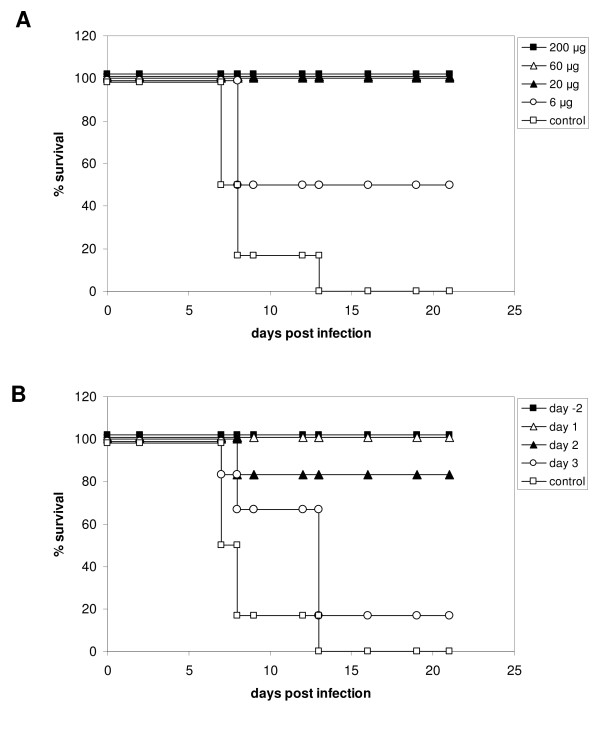
**Dose response and therapeutic activity of scFv-msFc-γ2c D005**. (A) Dose titration. Mice were treated with the indicated amounts of antibody on day -2, infected with Influenza A virus PR8 on day 0, and survival was monitored for 21 days. (B) Therapeutic setting. Mice were infected with Influenza A virus PR8 on day 0, treated with 200 μg of the antibody on the indicated days, and survival was monitored for 21 days. Control, 200 μg mouse IgG on day -2.

We next set out to determine the amount of antibody required to achieve protection in a prophylactic setting in mice. To this end, groups of mice were treated with decreasing amounts of scFv-msFc-γ2c D005 or, as a control, with 200 μg of mouse IgG. Two days later, animals were infected with a lethal dose of m.a. influenza A/PR/8/34 and monitored for 21 days (Figure 4A). As expected, control mice quickly succumbed to the infection and all had to be euthanized due to the severity of the disease within less than two weeks after challenge. In contrast, all animals that had received at least 20 μg of D005 antibody survived the challenge. Even at the lowest dose of 6 μg D005, half of the mice recovered and survived infection, indicating that the M2-specific antibody is a potent prophylactic agent.

### Therapeutic activity of M2e-specific antibody D005

In view of the potent prophylactic activity of D005, the antibody's ability to control an established viral infection was tested next (Figure 4B). Thus, groups of mice were infected with a lethal dose of m.a. influenza A/PR/8/34 and treated with a single injection of 200 μg scFv-msFc-γ2c D005 1, 2 or 3 days after challenge. Animals treated with 200 μg D005 or mouse IgG two days prior to the infection served as positive and negative controls respectively. In accordance with the results above all mice treated with the control antibody succumbed to the disease, whereas all mice that had received the D005 antibody prophylactically survived the lethal challenge. Importantly, almost all animals which had been treated therapeutically within two days after infection survived the challenge. Even animals that had been treated 3 days post infection appeared to withstand the infection longer than the control group.

Recently, the M2-specific human antibody Z3G1 has been reported to show therapeutic activity in mice [22]. However, multiple injections of Z3G1 starting as early as 5 h after infection were required for full protection. Antibody D005 compares favorably to this, since it was fully protective with a single injection at day 1 and still showed some protection when administered 3 days after challenge. Taken together, these data indicate that treatment with antibody D005, in addition to its potential as pre-exposure prophylaxis, may also be used therapeutically at the early stages of infection.

### Prophylactic activity of fully human IgG1k-D005

**Figure 5:**
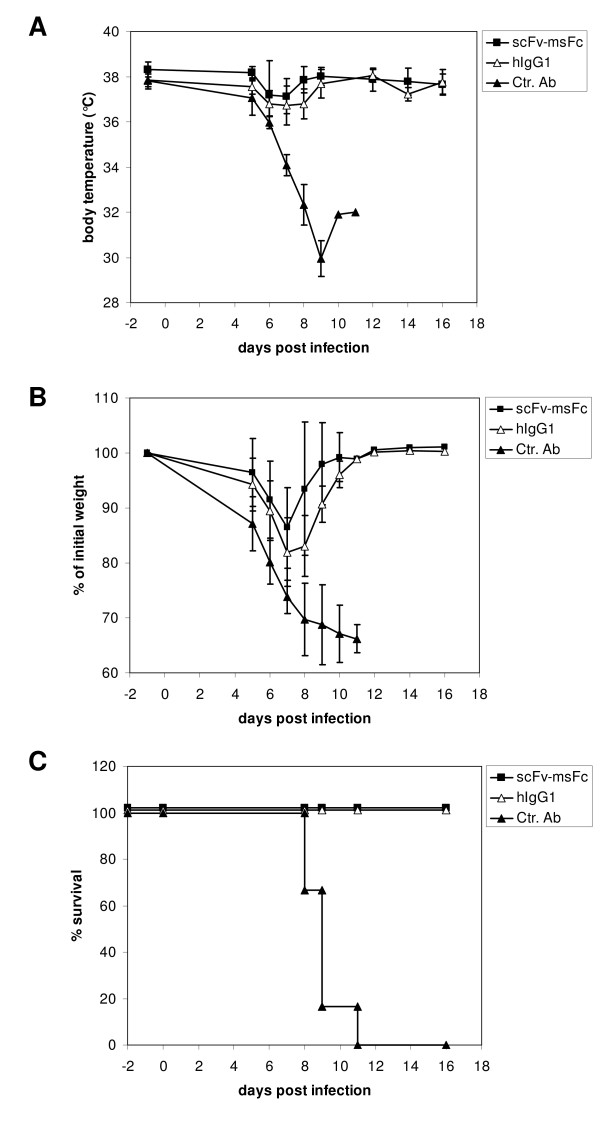
**Effect of M2-specific antibody D005 on Influenza-induced morbidity and mortality**. Mice were treated with 144 μg scFv-msFc-γ2c, 200 μg hIgG1 or 200 μg control human IgG (Ctr. Ab) on day -2, infected with Influenza A virus PR8 on day 0, and body temperature (A), weight (B) and survival (C) were monitored on the indicated days.

Given the intended use of the M2e-specific antibody in humans, D005 was produced as a fully human IgG1 in 293T cells using an EBV-based episomal expression system. Similar to the scFv-msFc-γ2c antibodies, IgG1-D005 was expressed at high levels in mammalian cells and yielded approximately 70 mg per liter culture supernatant. To evaluate its protective activity in the influenza A mouse model, IgG-D005 was compared to its scFv-msFc-γ2c counterpart. Thus, groups of mice were treated with equimolar amounts of IgG1-D005, scFv-D005-msFc-γ2c, or human IgG. Two days later, animals were infected with a lethal dose of influenza A/PR/8/34 and closly monitored for 16 days (Figure 5). Mice treated with control human IgG displayed a dramatic drop in body temperature and weight loss and had to be euthanized between 8 and 11 days after infection due the severity of the symptoms. In contrast, all animals treated with M2-specific antibodies survived the lethal challenge, hardly experienced any fever and showed only a transient drop in body weight. Thus, D005 was protective both as a scFv-msFc-γ2c fusion protein and as a fully human IgG1, indicating that the human antibody was able to recruit effector functions in the mouse system and that IgG-D005 may be used as a valuable prophylactic or therapeutic agent in humans.

## Conclusions

Human antibodies specific for the Influenza A M2 protein were isolated and shown to have potent prophylactic and therapeutic activity in a mouse model. This underscores the utility of Sindbis-based mammalian cell display for the isolation of natural human antibodies with therapeutic potential.

## Methods

### Identification of M2-specific antibodies by mammalian cell display

Peripheral blood mononuclear cells (PBMC) were isolated from 10 ml of heparinized blood by Ficoll gradient. PBMC were pre-incubated with Alexa 647 nm-labeled Qβ and human gamma globulin (Jackson ImmunoResearch) and then stained with: (1) M2e coupled to Qβ in combination with a Alexa 488 nm-labeled Qβ-specific mouse mAb, as well as the M2-specific mouse mAb 14C2 (Abcam) in combination with FITC-labeled donkey anti-mouse IgG antibody (Jackson ImmunoResearch); (2) PE-labeled mouse anti-human IgM, mouse anti-human IgD, mouse anti-human CD14, and mouse anti-human CD3 antibodies (all BD Biosciences/Pharmingen); and (3) PE-TexasRed-labeled mouse anti-human CD19 antibody (Caltag Laboratories). After staining, cells were washed and filtered, and 334 M2-specific B cells (FL1-positive, FL2-negative, FL3-positive, FL4-negative) were sorted on a FACSVantage^® ^SE flow cytometer (Becton Dickinson).

A Sindbis virus-based scFv cell surface display library was produced from antigen-specific B cells as described [33]. BHK cells were infected with the Sindbis library and cells displaying M2-specific scFv antibodies were isolated using M2e coupled to RNase A in combination with an RNase-specific rabbit polyclonal antibody (Abcam) and a FITC-labeled donkey anti-rabbit IgG antibody (Jackson ImmunoResearch). Alternatively, cells displaying M2-specific scFv antibodies were isolated using Qβ-M2e in combination with M2-specific mouse mAb 14C2 (Abcam) and FITC-labeled donkey anti-mouse IgG antibody (Jackson ImmunoResearch). Each cell was sorted into a well of a 24-well plate containing 50% confluent BHK feeder cells. Upon virus spread (2 days post sorting), the infected cells were tested by FACS analysis for M2-binding to identify virus clones encoding M2-specific scFv antibodies.

### Expression and purification of scFv antibodies

Fusion proteins were generated carrying an N-terminal human scFv fused to a C-terminal mouse Fc-γ2c domain. Thus, scFv coding regions were PCR amplified from Sindbis virus-containing supernatants by RT-PCR, digested with the restriction endonuclease Sfi1 and cloned into the expression vector pCEP-SP-Sfi-msFc-γ2c. This vector is a derivative of the episomal mammalian expression vector pCEP4 (Invitrogen), carrying the Epstein-Barr Virus replication origin (oriP) and nuclear antigen (encoded by the EBNA-1 gene) to permit extrachromosomal replication, and contains a puromycin selection marker in place of the original hygromycin B resistance gene. The resulting plasmids drive expression of scFv-msFc-γ2c fusion proteins under the control of a CMV promoter.

Fully human γ1 heavy chain and κ light chain coding regions were generated by total gene synthesis (GeneArt AG, Regensburg, Germany) and combined into the EBNA-based expression vector pCB15 essentially as described [33].

Expression of the scFv-msFc-γ2c fusion proteins, as well as fully human IgG1κ antibody was done by transfecting the expression vectors into HEK-293T cells, using Lipofectamin Plus (Invitrogen). For large scale production and purification, stable protein-expressing cells were enriched by selection in the presence of 1 μg/ml puromycin (Sigma). Pools of resistant cells were maintained in serum-free medium on Poly-L-Lysine coated dishes or in roller bottles for up to 3 weeks. Supernatants containing the respective antibodies were collected twice a week and filtered through a 0.22 μM Millex GV sterile filter (Millipore). Both types of antibodies were purified by affinity chromatography over a protein A-Sepharose column (GE healthcare).

### ELISA analysis

ELISA plates (96 well MAXIsorb, NUNC) were coated with RNAse-M2e-cons or RNAse-M2e-VN at a concentration of 4 μg/ml in coating buffer (0.1 M NaHCO3, pH 9.6) for one hour at 37°C. The plates were then washed with wash buffer (PBS/0.05% Tween) and blocked for 2 h at 37°C with 3% BSA in wash buffer. The plates were then washed again and incubated with 3-fold serial dilutions of scFv-msFc-γ2c in wash buffer containing 1% BSA. Plates were incubated for 2 h at room temperature and then extensively washed with wash buffer. Specifically bound antibodies were then detected with HRPO-labeled, Fcγ-specific, goat anti-mouse IgG antibody (Jackson ImmunoResearch Laboratories). After extensive washing with wash buffer, plates were developed with a 0.4 mg/ml solution of 1, 2-ortho-phenylenediamine dihydrochloride (OPD) in citric acid buffer (35 mM citric acid, 66 mM Na_2_HPO_4_, pH 5.0) containing 0.01% H_2_O_2_. After 10 min the reaction was stopped with a 5% solution of H_2_SO_4 _in H_2_O, and plates were read at 450 nm on an ELISA reader (Biorad Benchmark).

### Affinity measurement by Friguet ELISA

A 10 ng/ml solution of, respectively, scFv-D005-msFc-γ2c, scFv-E040-msFc-γ2c or scFv-F052-msFc-γ2c, was incubated in the presence of different concentrations of M2e-cons peptide (3-fold serial dilutions corresponding to 10 nM to 0.17 pM) in PBS/1% BSA. After 2 h at room temperature, free antibody was detected by a classical ELISA similar to the one described above. For this, ELISA plates that had been coated with RNAse-M2e-cons conjugate at a concentration of 20 ng/ml at 4°C overnight were washed with wash buffer (PBS/0.05% Tween) and blocked for 2 h at 37°C with 3% BSA in wash buffer. The plates were then washed again and incubated with the solution binding reactions for 30 min at room temperature. After extensive washing with wash buffer, bound scFv-Fcγ2c fusion proteins were detected by a 1 h incubation at room temperature with a HRPO-labeled, Fcγ-specific, goat anti-mouse IgG antibody (Jackson ImmunoResearch Laboratories). After extensive washing with wash buffer, plates were developed as described above. The Kd values were determined as the EC50 of the ELISA signal as a function of the M2e-cons peptide concentration present in the solution binding reaction.

### Mouse model of Influenza A infection

Prophylactic and therapeutic activity of antibodies was tested in a mouse model of Influenza A infection. Thus, six weeks old female C57BL/6 mice (6 per group) were infected intranasally with a lethal dose of mouse-adapted (m.a.) influenza A virus PR8 (4 × LD50), followed by close monitoring of weight-loss and fever (twice daily at peak of infection). Antibodies were injected intraperitoneally either 2 days before (prophylactic setting) or 1 to 3 days after infection (therapeutic setting). One day later, mice were bled in order to verify the presence of the antibodies in the blood by ELISA (not shown). Antibodies were readily detectable in the sera of all mice, except for one mouse receiving 500 μg scFv-D005-msFc-γ2c (Figure 3) and one mouse receiving 60 μg scFv-D005-msFc-γ2c (Figure 4A); these mice were subsequently removed from the analysis. All animal experiments were carried out in accordance with protocols approved by the Swiss Federal Veterinary Office. Animals whose body temperature reached 30°C were considered moribund and had to be euthanized immediately due to abortion criteria defined by the Veterinary Office.

## Competing interests

All authors are present or former employees of Cytos Biotechnology AG and hold stocks or stock options in the company. The authors have no additional financial interests.

## Authors' contributions

RRB, MB, NS, WAR, PS, and MFB designed research; RRB, MB, RBB, MG, and SM performed research; RRB, MB, NS, WAR, PS, and MFB analyzed data; RRB wrote the paper. All authors read and approved the final manuscript.
